# Cytokine signatures associate with disease severity in children with *Mycoplasma pneumoniae* pneumonia

**DOI:** 10.1038/s41598-019-54313-9

**Published:** 2019-11-28

**Authors:** Mingyue Yang, Fanzheng Meng, Man Gao, Genhong Cheng, Xiaosong Wang

**Affiliations:** 1 Department of Translational Medicine, The First Hospital of Jilin University, Changchun, China; 2 Department of Pediatrics, The First Hospital of Jilin University, Changchun, China; 3 Department of Microbiology Immunology and Molecular Genetics, University of California Los Angeles, Los Angeles, USA

**Keywords:** Respiratory tract diseases, Acute inflammation

## Abstract

Host immune response may be involved in the pathogenesis of children *Mycoplasma pneumoniae* pneumonia (MPP). In the current study, we investigated the alterations of cytokines levels among control, mild MPP and severe MPP children to determine whether cytokine signatures associate with MPP and correlate with disease severity. We measured 13 cytokines in bronchoalveolar lavage fluid (BALF) of 88 children with MPP and 26 children with foreign body aspiration (FB) using a Luminex system. Linear discriminant analyses were performed to develop predictive models of mild MPP and severe MPP on these children. We observed nearly complete separations of severe MPP group, mild MPP group and control group in linear discriminant analyses. Eleven cytokines significantly increased in children with MPP, and seven cytokines had statistically significant upward linear trends correlated with MPP severity. In addition, compared to control group, both IFNγ/IL4 ratio and IFNγ/IL13 ratio increased in mild MPP and severe MPP groups. Our results suggest that children MPP can alter BALF cytokines signatures which associate with disease severity and can be characterized by a distinct airway molecular phenotype that has elevated Th1/Th2 ratios.

## Introduction

*Mycoplasma pneumoniae* (MP) is one of the most common pathogens in children’s community-acquired pneumonia (CAP)^1,2^. *Mycoplasma pneumoniae* pneumonia (MPP) is generally self-limited and can be effectively controlled by macrolides^3^. However, severe MPP often happens and has complications such as pleural effusion, atelectasis and lung consolidation. According to literatures and our clinical experience, additional corticosteroids can effectively improve the clinical symptoms and chest radiographic manifestations of these severely affected children^4–6^. Although the severity of the disease appears to be related to the degree of the host immune response to the infection^7^, the pathogenesis of MPP is still not clear.

The purpose of the current study is to determine whether an abnormal profile of cytokines can be identified in MPP children and whether this profile correlates with disease severity. Bronchoalveolar lavage fluid (BALF) was derived from the lung tissue surface of the lesion, which directly reflects the inflammation and immunological responses of the infected local lung tissue. Linear discriminant analysis is used here to discriminate severe MPP from mild MPP and healthy control in children. It is a supervised method of high-dimensional data reduction on the levels of cytokines measured in BALF. These cytokines were selected by their biological relevance to MPP or immune cells involved in lung infection (see Supplementary Table S1). Eleven cytokines were found higher in MPP children compared with controls. Seven cytokines can be closely correlated with MPP severity and five of them are proinflammatory cytokines that may contribute to many of the symptoms these children experience. Further characterization of immune alterations in BALF of children MPP may provide additional clinical insight in the pathogenesis of the disease.

## Results

### Basic data of the clinical samples

Children’s clinical information was listed in Table 1. All of the severe MPP children and 16 mild MPP children had lobar pneumonia. Twenty-nine mild MPP children had segmental pneumonia. Eighteen FB children were enrolled during the treatment of bronchus foreign body-removing, which was on the same day of foreign body aspiration. Eight FB children were enrolled during re-examination, which was about one week after bronchus foreign body removing. Children of the FB group were significantly younger that of the MPP groups. Fever duration, C-reactive protein (CRP), blood neutrophil count and blood lymphocyte count are significantly different between mild MPP group and severe MPP group.

**Table 1:** Clinical characteristics of the children involved in the current study.

	Control	Mild MPP	Severe MPP	Control vs. Mild	Control vs. Severe	Mild vs. Severe
	(n = 26)	(n = 45)	(n = 43)	p value	p value	p value
Gender (female/male)	8/18	22/23	13/30	0.1411	0.969	0.0763
Median age (range), years	1.83 (0.92–3)	5 (1.67–12)	6 (1.75–13)	<0.0001^***^	<0.0001^***^	0.9162
Fever duration, days	—	8.84 ± 4.67	10.58 ± 4.23	—	—	0.0089^**^
Glucocorticoid (yes/no)	0/26	7/38	27/16	0.0365^*^	<0.0001^***^	<0.0001^***^
Pleural effusion (yes/no)	0/26	2/43	11/32	0.5292	0.0048^*^	0.0056^**^
Extrapulmonary manifestations (yes/no)	0/26	0/45	2/41	>0.9999	0.2772	0.3458
Median CRP (range), mg/L	—	19.04 ± 23.96	34.57 ± 40.1	—	—	0.0057^**^
Peripheral blood cell count						
Blood leukocyte count (×10^9^ cells/L)	—	8.59 ± 3.17	9.6 ± 4.83	—	—	0.7134
Blood monocyte count (%)	—	8 (3–13)	7 (2–12)	—	—	0.3683
Blood neutrophil count (%)	—	59 (20–86)	68 (48–89)	—	—	0.0031^**^
Blood lymphocyte count (%)	—	29 (11–70)	22 (9–39)	—	—	0.0021^**^
Blood eosinophil count (%)	—	2 (0–5)	2 (0–5)	—	—	0.7268
Indication of bronchoscopy						
Diagnosis and remove of bronchus foreign body	18	0	0	—	—	—
Re-examination of bronchus foreign body	8	0	0	—	—	—
Diagnosis and treatment of lobar pneumonia	0	16	43	—	—	—
Diagnosis and treatment of segmental pneumonia	0	29	0	—	—	—
Timing of BALF collection						
Before treatment	26	45	43	—	—	—
After the treatment	0	0	0	—	—	—

Quantitative data with a normal distribution are presented as mean ± SD. Quantitative data with a non-normal distribution are presented as median (IQR).

p value: *p < 0.05, **p < 0.01, ***p < 0.001.

### Cytokine concentrations and linear discriminant analysis

The protein concentrations of TNFα, IL6, IL1β, MCP1, IL4, IL10, IFNγ, IL13, IL5, sCD40L, Flt3L, IL2 and IFNα2 in BALF samples were measured using a Luminex system (Table 2).

**Table 2:** Cytokine concentrations (pg/ml) in the bronchoalveolar lavage fluid.

	Control (n = 26)	Mild MPP (n = 45)	Severe MPP (n = 43)
TNFα	8.65 ± 2.32	80.31 ± 17.79	159.36 ± 27.88
IL6	34.67 ± 11.32	123.23 ± 29.32	348.09 ± 73.86
IL1β	23.3 ± 10.51	103.93 ± 27.8	408.66 ± 73.89
MCP1	310.95 ± 45.42	1820.35 ± 281.8	3173.96 ± 377.59
IL4	5.43 ± 1.05	15.12 ± 2.81	31.82 ± 4.92
IL10	1.96 ± 0.35	33.71 ± 7.05	75.54 ± 17.99
IFNγ	2.54 ± 0.36	26.55 ± 6.21	57.06 ± 12.46
IL13	9.09 ± 1.52	17.97 ± 1.7	21.68 ± 1.91
IL5	0.25 ± 0.04	3.29 ± 0.82	9.7 ± 3.86
sCD40L	8.33 ± 2.55	32.84 ± 4.85	36.66 ± 4.57
Flt3L	9.46 ± 0.79	19.81 ± 1.21	24.28 ± 1.24
IL2	0.64 ± 0.16	1.04 ± 0.29	2.04 ± 0.42
IFNα2	14.33 ± 2.46	17.97 ± 1.45	28.12 ± 2.26

Data represent the mean ± S.E.M.

**Figure 1:**
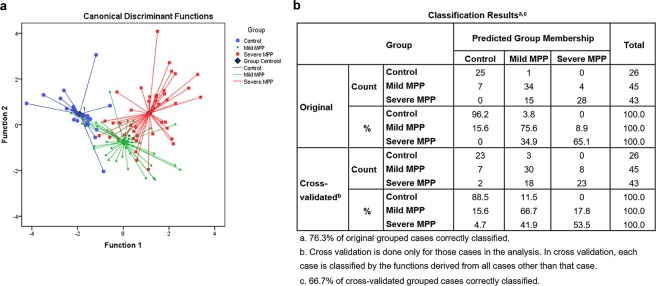
Linear discriminant analysis results. (**a**) Scatterplot of the discriminant functions generated from 13 factors. Each data point represents a single BALF sample. The plot depicts clustering and clear separation of severe MPP children (red square), mild MPP children (green triangles) and FB children (blue circles). Dark blue diamond represents group centroid. (**b**) Classified accuracy of the original grouped cases and cross-validated grouped cases were shown as well.

Linear discriminant analysis was performed to differentiate children with FB, mild MPP and severe MPP (Fig. 1a). Using the variances from all the values, two discriminant functions were derived, which accounted for 100% of the variance of the dataset. Results showed good separations of the three groups. Discriminant analysis results are shown in Fig. 1b. Original group method can correctly identify 96.2% of controls, 75.6% of mild MPP children and 65.1% of severe MPP children. Cross-validated group method can correctly identify 88.5% of controls, 66.7% of mild MPP children and 53.5% of severe MPP children. Stepwise discriminant analysis revealed that Flt3L, IFNα2 and IL1β contributed the most to the differentiation of mild MPP, severe MPP, and FB children. These three cytokines distinguished the three groups with an accuracy of 72.8% (an accuracy of 71.9% with cross-validation analysis).

### Cytokines increased in MPP groups

We further compared the protein concentrations between every two groups using statistical analysis. The p values before and after BH adjustment were listed in Table 3. Firstly, eleven cytokines rose in both mild MPP children and severe MPP children. Secondly, seven cytokines of TNFα, IL6, IL1β, MCP1, IL4, IL10, and IFNγ significantly increased in mild MPP children compared to controls and they increased further in severe MPP children. Gradual increases of these cytokines appeared in all the groups and these changes were also significant after BH adjustment. Thirdly, four cytokines of IL13, IL5, sCD40L and Flt3L remarkably increased in both mild MPP children and severe MPP children compared to controls. However, their increases were similar in severe MPP and mild MPP children. Lastly, no significant differences of IL2 and IFNα2 levels were found in mild MPP children compared to controls. However, both of them significantly increased in the severe MPP children, which may help to distinguish severe MPP from mild MPP and control.

**Table 3:** The p values before and after the adjustment for multiple comparisons.

Cytokine	Control vs. Mild MPP		Control vs. Severe MPP		Mild MPP vs. Severe MPP	
	p value	adjusted p value	p value	adjusted p value	p value	adjusted p value
TNFα	<0.0001	0.0002	<0.0001	0.0002	0.0002	0.0004
IL6	0.0002	0.0004	<0.0001	0.0002	0.0017	0.0022
IL1β	0.0004	0.0007	<0.0001	0.0002	<0.0001	0.0002
MCP1	<0.0001	0.0002	<0.0001	0.0002	0.005	0.0063
IL4	0.0007	0.0011	<0.0001	0.0002	0.0002	0.0004
IL10	<0.0001	0.0002	<0.0001	0.0002	0.01	0.0118
IFNγ	<0.0001	0.0002	<0.0001	0.0002	0.0068	0.0083
IL13	0.001	0.0013	<0.0001	0.0002	0.1362	0.1476
IL5	0.0006	0.0009	0.0002	0.0004	0.1822	0.0004
sCD40L	<0.0001	0.0002	<0.0001	0.0002	0.2425	0.2489
Flt3L	0.0008	0.0011	<0.0001	0.0002	0.0923	0.1028
IL2	0.5555	0.5555	0.0005	0.0008	0.0004	0.0007
IFNα2	0.0567	0.0650	<0.0001	0.0002	0.0008	0.0011

The p values have been adjusted by Benjamini-Hochberg procedure.

**Figure 2:**
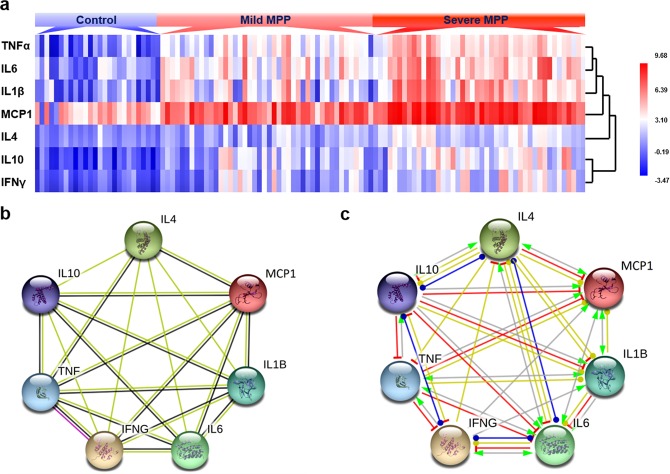
Analysis of the expression levels of TNFα, IL6, IL1β, MCP1, IL4, IFNγ and IL10 in BALF. (**a**) Cluster analysis results showing representative expression patterns in each group. (**b**) Diagram of protein protein interaction (PPI) network showing the evidence of the relationships among the proteins. The known or predicted 3d structure of the protein was shown inside the circles. The types of interaction evidence were indicated as below: yellow line represents the text mining evidence; black line represents the co-expression evidence and purple line represents the experimental evidence. (**c**) Diagram of PPI network showing type of interactions among the proteins. Arrow line represents positive action, T-type line represents negative action and round head line represents unspecified action. Yellow line means transcriptional regulation, red line means inhibition, blue line means binding and gray line means reaction.

It is interesting to notice that the concentrations of TNFα, IL6, IL1β, MCP1, IL4, IL10 and IFNγ increased significantly in mild MPP and further increased significantly in severe MPP, which indicate that these proteins may play important roles in the incidence and severity of children MPP. To compare the expression patterns of these proteins, we analyzed the results using cluster analysis (Fig. 2a). The expression patterns confirmed higher expression levels in mild MPP, the highest expression levels in severe MPP group and the lowest expression levels in control group. MCP1 levels are the highest among all the cytokines. The expression pattern of TNFα is close to IL6, while IL1β, MCP1 and IL4 have similar expression patterns as TNF and IL6. The expression patterns of IL10 and IFNγ are very close to each other.

To further explore the interactions among TNFα, IL6, IL1β, MCP1, IL4, IL10 and IFNγ, we analyzed these cytokine-levels using STRING database. Close interactions were found among these cytokines (Fig. 2) and Fig. 2b showed the evidence of the interactions. Text mining evidence (yellow line) can be found between any two of these cytokines. TNFα and MCP1 interacted with other cytokines based on the co-expression evidence (black line). Experimental evidence can also be found between TNFα and IFNγ (purple line). Figure 2c shows the close molecular action network between every two cytokines. Most of them are transcriptional regulations (yellow line) and only some of them had protein-protein reaction (gray line). The interactions between some cytokines are not specified, which can be positive or negative actions under different situations.

### Th1/Th2 balance in children with MPP

**Figure 3:**
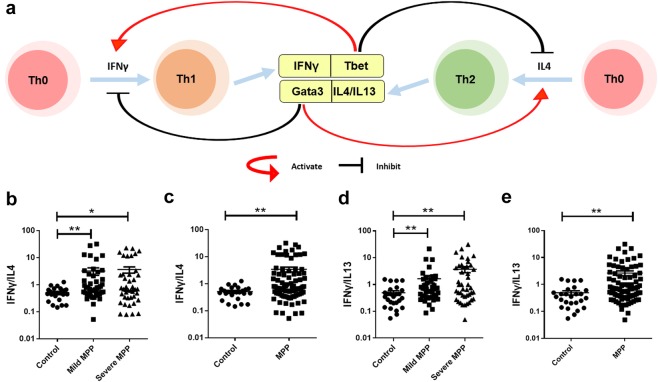
The analysis of Th1/Th2 ratios in MPP children. (**a**) Model diagram showing Th cell differentiation. (**b**) The ratios of IFNγ/IL4 in control, mild MPP and severe MPP groups. (**c**) The ratios of IFNγ/IL4 in control group and MPP group. (**d**) The ratios of IFNγ/IL13 in control, mild MPP and severe MPP groups. (**e**) The ratios of IFNγ/IL13 in control group and MPP group. The results of Benjamini-Hochberg adjusted p were shown as: *padj < 0.05, **padj < 0.01, ***padj < 0.001.

Debates about the inflammatory factors in MPP patients focus on the Th1/Th2 balance^8,9^. Figure 3a shows how Th1 related factor IFNγ and Th2 related factor IL4 affect the differentiation of Th1 and Th2 cells, as well as the balance between Th1 and Th2 cells. IFNγ and Tbet promote the differentiation of Th0 to Th1 and IL4 and Gata3 promote the differentiation of Th0 to Th2. To explore the Th1/Th2 balance in BALF of MPP children, the ratios of IFNγ/IL4 were calculated (Fig. 3b,c). Ratios of IFNγ/IL4 in both mild MPP group and severe MPP group rose significantly compared to control group (Fig. 3b, p_adj_ < 0.05) while there was NO significant difference between mild MPP and severe MPP groups. We combined the results of mild MPP group and severe MPP group as the results of MPP group. The IFNγ/IL4 ratios significantly increased in MPP group compared to control (Fig. 3c, p_adj_ < 0.01). As IL13 is a Th2 related cytokine expressed in lung, the ratios of IFNγ/IL13 were also calculated. Similarly, the ratios of IFNγ/IL13 in mild MPP group and in severe MPP group rose significantly compared with control group (Fig. 3d, p_adj_ < 0.01) while there was NO significant difference between mild MPP group and severe MPP group. IFNγ/IL13 ratios significantly increased in MPP group compared to control (Fig. 3e, p_adj_ < 0.01). These results show that the increase of Th1 related cytokine (IFNγ) is much higher than the increase of Th2 related cytokines (IL4 and IL13) in both mild MPP and severe MPP children.

**Figure 4:**
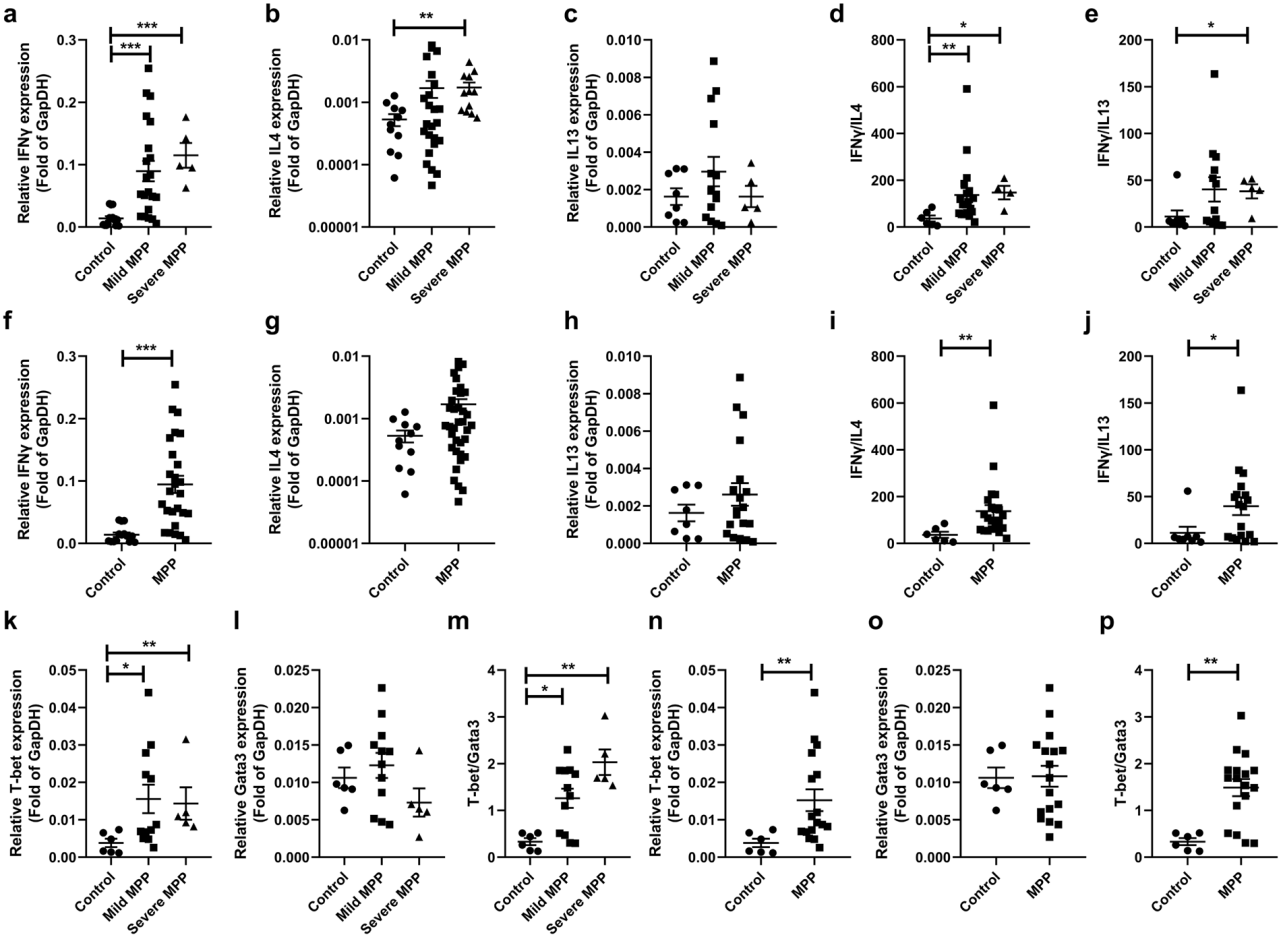
mRNA levels of IFNγ, IL4, IL13, T-bet and Gata3 in BALF of children with MPP. (**a**) mRNA levels of IFNγ in control, mild MPP and severe MPP groups. (**b**) mRNA levels of IL4 in control, mild MPP and severe MPP groups. (**c**) mRNA levels of IL13 in control, mild MPP and severe MPP groups. (**d**) The ratios of IFNγ/IL4 in control, mild MPP and severe MPP groups. (**e**) The ratios of IFNγ/IL13 in control, mild MPP and severe MPP groups. (**f**) mRNA levels of IFNγ in control and MPP groups. (**g**) mRNA levels of IL4 in control and MPP groups. (**h**) mRNA levels of IL13 in control and MPP groups. (**i**) The ratios of IFNγ/IL4 in control and MPP groups. (**j**) The ratios of IFNγ/IL13 in control and MPP groups. (**k**) mRNA levels of T-bet in control, mild MPP and severe MPP groups. (**l**) mRNA levels of Gata3 in control, mild MPP and severe MPP groups. (**m**) The ratios of T-bet/Gata3 in control, mild MPP and severe MPP groups. (**n**) mRNA levels of T-bet in control and MPP groups. (**o**) mRNA levels of Gata3 in control and MPP groups. (**p**) The ratios of T-bet/Gata3 in control and MPP groups. p values were shown as: *p < 0.05, **p < 0.01, ***p < 0.001.

To further confirm these findings, we measured the mRNA levels of related factors (Fig. 4). mRNA levels of IFNγ in mild and severe MPP groups were significantly higher than that of control group (p < 0.001, Fig. 4a). However, there was NO significant difference between severe MPP group and mild MPP group. Compared to control group, IL4 levels increased greatly (p = 0.0035) in severe MPP group (p < 0.01, Fig. 4b) and IL13 levels of mild MPP and severe MPP groups didn’t change (Fig. 4c). The ratios of IFNγ/IL4 in mild MPP and severe MPP groups rose significantly compared to control group (p < 0.05, Fig. 4d). The IFNγ/IL13 ratios significantly increased in severe MPP group compared with control group (p < 0.05, Fig. 4e). IFNγ levels in MPP group were greatly higher than that in control group (p < 0.001, Fig. 4f). The ratios of IFNγ/IL4 and IFNγ/IL13 increased significantly in MPP group (Fig. 4i,j, p < 0.05). However, IL4 (Fig. 4g) and IL13 (Fig. 4h) levels didn’t change in MPP group.

On the other hand, we measured the mRNA levels of the transcription factors T-bet and Gata3 (Fig. 4k–p). T-bet levels in mild MPP and severe MPP groups rose significantly compared to that of control group (p < 0.05, Fig. 4k). Gata3 levels of mild MPP and severe MPP groups didn’t change (Fig. 4l). The ratios of T-bet/Gata3 in mild MPP and severe MPP groups increased greatly compared to control group (p < 0.05, Fig. 4m). T-bet levels in MPP group were also significantly higher than that in control group (p < 0.01, Fig. 4n). Gata3 levels didn’t change in MPP group (Fig. 4o). T-bet/Gata3 ratios rose significantly in MPP group (p < 0.01, Fig. 4p).

## Discussion

Through multiplex approach, we measured 13 cytokines in BALF of 45 mild MPP children, 43 severe MPP children and 26 FB children as control. To the best of our knowledge, there is no previous study reporting multiplex results of the cytokines in BALF of children with MPP. The main finding of this study is that 13 cytokines helped to separate children with FB, mild MPP or severe MPP. Eleven cytokines increased greatly in both mild MPP children and severe MPP children. Significantly elevated trend of TNFα, IL6, IL1β, MCP1, IL4, IFNγ and IL10 was identified in mild MPP and severe MPP. Both Th1 related factor and Th2 related factors increased in the BALF of MPP children, while the ratios of IFNγ/IL4 and IFNγ/IL13 significantly rose in MPP children compared to control children. These results support a Th1-type dominant local response in the airway of acute children MPP.

We did discriminant analysis on the 13 cytokine levels in BALF. The linear combination of these cytokine-levels led to clear separations of severe MPP, mild MPP and control groups. Thus, we conclude that unique patterns of airway inflammations associate with mild MPP and severe MPP in children. The pathogenesis of severe MPP is complex and not completely understood. Linear discriminant analyses reveal relatively discrete groupings of control, mild MPP and severe MPP groups, which suggest that mild and severe MPP are truely phenotypically distinct.

Seven cytokines of TNFα, IL6, IL1β, MCP1, IL4, IFNγ and IL10 increased significantly in mild MPP group compared to control group, and further markedly increased in severe MPP group compared to mild MPP group. Even after corrections for multiple comparisons, linear relationships between these cytokine levels with disease severity are still statistically significant. No previous reports are found to apply the high-dimensional data reduction techniques on inflammatory cytokine study in BALF of children with MPP. However, ELISA is also a reliable method and has been used to study the cytokine levels in MPP children^8–11^. We compared published ELISA results with our Luminex assay results. Similar changes on some cytokine levels are reported in previous publications. For example, TNFα levels increased in the peripheral blood of patients with MPP^12^ and severe MPP^13^ as well as those with refractory MPP^14^. Increased levels of TNFα is related to acute MPP^11^, refractory MPP^10^ and wheezy in children with MPP^15^. TNFα is also an optimal predictor for segmental MPP or lobar MPP with pleural effusion^13^. Another research team also reported significantly elevated levels of IL6 in the BALF of children with severe MPP compared to control^16^. IL6 levels in refractory MPP children are higher than those in general MPP children^17^. Xu’s team reported that serum TNFα, IL6, IFNγ and IL10 levels of MPP group are significantly higher than control group^18^.

Increased levels of IL4 were reported in the BALF of children with MPP^19^, which were related to their high-MP-load^20^. On the contrary, another team didn’t find significant change in the levels of IL4 in the serum of children with MPP^21^. Some research teams reported increased levels of IFNγ in the serum of MPP children^12^ and refractory MPP children^14,17^. However, one team reported decreased IFNγ in serum of MPP children than control^15^, another group reported that IFNγ levels in BALF of MPP children is not different from that of the control children^19^. Zhao *et al*. found significantly higher levels of IL4 and IFNγ in the serum of children with segmental MPP or lobar MPP than those of children with bronchial MPP^13^. Results reported so far have been controversial whether IL4 and IFNγ increase in MPP children. Our current study provide evidences to the significant increases of IL4 and IFNγ in mild MPP children and severe MPP children. With regard to IL10, similar to our results, some teams reported significantly increased levels of IL10 in the serum of MPP children^12,15,21^, which further rose in refractory MPP children^17^ and severe MPP children^13^. Guo’s team found IL10 increased in BALF of MPP children^16^, which is consistent with our findings.

We provided the first preliminary evidence of the molecular phenotype of children severe MPP using the supervised method of linear discriminant analysis. In terms of pathogenesis, previous research focused on the roles of TNFα and IFNγ. Increased levels of TNFα were related to many pulmonary inflammatory diseases including chronic obstructive pulmonary disease (COPD), acute respiratory distress syndrome (ARDS), acute lung injury (ALI), sarcoidosis, asthma and interstitial pulmonary fibrosis (IPF). People find TNFα playing multiple roles in disease pathology, which include stimulating the generation of inflammatory mediators, inducing the accumulation of inflammatory cells and causing airway hyperresponsiveness^22^. The proinflammatory cytokine IFNγ is well known for its major roles in innate immunity and adaptive immunity against intracellular infections. IFNγ can coordinate a diverse array of cellular programs through its transcriptional regulation of the immunologically relevant genes. The cellular effects of IFNγ include up-regulating the antigen presentation, pathogen recognition, leukocyte trafficking and immunomodulation^23^. IL10 has broad anti-inflammatory properties, which include counteracting the function of Th1 cells. During Toxoplasma gondii infection, people discovered that IFNγ-producing Tbet (+) Foxp3 (−) Th1 cells produced host-protective IL10 in an autocrine manner, which played important roles of regulating immunopathology^24^.

IL13, which is highly expressed in the lung, possibly plays pivotal roles in the onset and exacerbation of human bronchial asthma^25^. As a classic feature of asthma, IL5 activates eosinophils and cause airway inflammation^26^. Increased levels of IL5 and IL13 found in MPP children may be connected to the concurrence of MPP and asthma in children. Some results show that MPP children with wheeze have significantly higher levels of IL5 and VEGF in the serum compared to MPP children without wheeze^27^. Flt3L is critical for instructing the generation of DC from Flt3+ lymphoid and myeloid-committed progenitors throughout different organs *in vivo*^28^. One team reported significantly increased IL2 levels in BALF of MPP children comparing to control^19^, which is consistent with our findings. We also found that the levels of IL1β, MCP1, sCD40L and IFNα increased significantly in MPP children, which has not been reported so far by other teams.

Limitations of the current study are worth discussing. First, because the children in FB control group are significantly younger than that of the MPP groups, they are not perfect controls. Although we didn’t see difference in cell composition between the groups^29^, immune response types may change as the children grow. The lack of a healthy pediatric control group because of ethical reasons does raise the question whether age affects the experimental results. Second, although the ratios of IFNγ/IL4, IFNγ/IL13 and T-bet/Gata3 may reflect the activation ratios of Th1/Th2 cells, the increased levels of IFNγ may also reflect the activation of other cells including ILCs, NKT cells and NK cells. Further studies will be needed to explore the existence and function of these cells in BALF of MPP children. The last is the cross-sectional design. Future longitudinal studies are necessary to address whether MPP children remain within their cytokine signature over time.

In summary, the current study identified a number of factors that increased in children with MPP, and discussed the role of multiple factors in MPP. We demonstrated a unique molecular phenotype of mild MPP and severe MPP characterized by increased BALF inflammation, which may provide support for the use of multiple discriminators for distinguishing severe MPP from mild MPP in children. We hope these findings will help better understanding of the severe MPP phenotype. Future studies will be required to further elucidate their clinical implications.

## Methods

### Clinical information

Eighty-eight children with MPP (mild MPP, n = 45; severe MPP, n = 43) and 26 children with foreign body aspiration (FB) hospitalized in the First Hospital of Jilin University from January 2016 to December 2017 are recruited in the current study. The diagnosis of MP infection is based on serologic test and confirmed by polymerase chain reaction (PCR). Mild community-acquired pneumonia and severe community-acquired pneumonia are defined based on the criteria described^30–32^. Mild is defined as respiratory rate <70 breaths/min at age <3 years old or respiratory rate <50 breaths/min at age ≥3 years old, normal food-intake, no dehydration^33^. Severe is defined as respiratory rate ≥70 breaths/min at age <3 years old or respiratory rate >50 breaths/min at age ≥3 years old, cyanosis, flaring of the nares, marked retractions, anorexia and dehydration; or MPP combined with pleural effusion, lung necrosis/lung abscess and other complications in lung; or MPP combined with dysfunction of other systems^34^. By measuring the maximum thickness of fluid between the visceral and parietal pleurae on a CT scan, pleural effusion was defined as fluid thickness >5 mm^35^. Children with other respiratory infections and tuberculosis are excluded in terms of the following tests: purified-protein derivative (PPD), blood culture, pleural effusion culture, nasopharyngeal suction/swab culture, nasopharyngeal suction/swab virus antigen detection and serology for *Chlamydia pneumonia* and *Legionella pneumonia*. Children, who had asthma, repeated respiratory infections, chronic heart disease, rheumatic diseases or immune deficiencies are also excluded. BALF samples are collected only from children with acute MPP before treatment. Children with mild MPP are only chosen when they hav segmental or lobular pneumonia. Children with FB are included as control, which had no respiratory tract illness in the previous 6 weeks. At the moment of sample collecting, the FB children had no clinical symptom, no sign of lung inflammation or infection observed under brochoscopy.

### Ethics approval

Ethical approval for this study was received from the Institutional Medical Ethics Review Board of the First Hospital of Jilin University (reference number: 2016–394) in compliance with the Declaration of Helsinki.

### Informed consent

Parents or legal guardians of all children provided written informed consents.

### Brochoscopy and bronchoalveolar lavage

Following the guidelines described earlier^30,33,36^, in order to diagnose and treat lobar pneumonia or segmental pneumonia, flexible fiber optic bronchoscopy with bronchoalveolar lavage was performed within three days after admission. BALF was gently aspirated, collected and centrifuged at 1500 rpm for 5 minutes at 4 °C within 1 hour after collection. The supernatant was divided into 300 µl aliquots and stored in −40 °C freezer.

### Cytokine assay

Cytokine levels were determined using a Human Cytokine/Chemokine Magnetic Bead Panel kit (HCYTOMAG-60K, Millipore, Billerica, MA) following the manufacturer’s protocol^37^. BALF samples and serial diluted standards were added to 96 well plates and incubated overnight in 4 °C. Each sample was added into two replicate wells. Then, plates were run on the Luminex 200™ machine with xPONENT 3.1 software. We utilized MILLIPLEX Analysis 5.1 software to calculate the assay sensitivities (minimum detectable concentrations, minDC, pg/mL) and analyze the Luminex assay results. The sensitivities of each cytokine were listed in Supplementary Table S2. The accuracy and precision of the kit were showed in Supplementary Table S3. Sample concentrations were determined using a 5-point logistic curve fitting algorithm in the software. Results were accepted as final if the coefficient of variation (CV) <10%. The CV of each children group can be found in Supplementary Table S4.

### Real-time quantitative PCR (qPCR)

We performed qPCR as described previously^33,38^. Briefly, we extracted total RNA and reverse-transcribed RNA using the PrimeScript RT Reagent Kit (TAKARA). cDNA was amplified using the Fast Start Universal SYBR Green Master (Roche Diagnostics GmbH, Germany) and analyzed with specific primers (synthesized by Sangon Biotech) on the Applied Biosystems Step one plus instrument (Step one software 2.2). We selected glyceraldehydes-3-phosphate dehydrogenase (GAPDH) as reference gene and used 2^−ΔΔCT^ method to analyze data, which was expressed as the fold-change relative to the average value of the GAPDH^39,40^.

### Data analysis

The linear discriminant analysis is a multivariate statistical analysis method, which uses various eigenvalues of a research to predict the group membership^41,42^. In order to provide the best discrimination between groups, this procedure yields a set of discriminant functions based on the linear combinations of variables. We plotted these functions to show the proximity of that subject to others in the same group. We performed cross-validation of the models by randomly selecting subject by a random number generator and repeating the analyses. Fisher method is used for the linear discriminant analysis^37,43^.

Statistical analyses were performed using Graphpad 5.0 or SPSS software (version 22.0). Mann-Whitney U test was used for comparison. The classification data were compared by chi-square test. Statistical significance was defined as p < 0.05. For the reanalyzed data sets, p values were adjusted by Benjamini-Hochberg (BH) procedure^44^ using R programming language 3.1.1. Adjusted p value was expressed as p_adj_, statistical significance was defined as p_adj_ < 0.05.

For comparison of the different matrices, we generated unsupervised heat maps by normalizing all data between maximum (red) and minimum (blue) for each individual concentration using Heml 1.0.3.3-Heatmap Illustrator. Protein-protein interaction (PPI) patterns were analyzed using STRING database (http://string-db.org/; version 10.5)^33,45^ with a confidence score >0.7.

## Supplementary information

Dataset 1

Dataset 2

Dataset 3

Dataset 4

## Data Availability

The datasets generated during and analysed during the current study are available from the corresponding author on reasonable request.
