# Supplementary material for: Cytokine signatures associate with disease severity in children with Mycoplasma pneumoniae pneumonia
Source: Sci Rep. 2019 Nov 28;9:17853. doi: 10.1038/s41598-019-54313-9 (PMC6882793; doi:10.1038/s41598-019-54313-9)
Supplement: Supplementary file 1 — Dataset 1 [file 41598_2019_54313_MOESM1_ESM.pdf]

Title: Cytokine signatures associate with disease severity in children with *Mycoplasma pneumoniae* pneumonia

Mingyue Yang[1], Fanzheng Meng[2], Man Gao[2], Genhong Cheng[3], Xiaosong Wang[1*]

* Correspondence: Xiaosong Wang

Postal address: No. 519 Dongminzhu Street, Chaoyang District, Changchun 130021, China

Tel: +86 138 4498 9650

Fax: +86 431 8565 4528

E-Mail: xiaosongwang@jlu.edu.cn

1. Department of Translational Medicine, the First Hospital of Jilin University, Changchun, China
2. Department of Pediatrics, the First Hospital of Jilin University, Changchun, China.
3. Department of Microbiology Immunology and Molecular Genetics, University of California Los Angeles, Los Angeles, USA

Table S1. Rationale of cytokine and chemokine selections

	Biological Actions Relevant to MPP	Reference
**TNFα**	TNFα plays multiple roles in disease pathology by inducing an accumulation of inflammatory cells, stimulating the generation of inflammatory mediators, and causing oxidative and nitrosative stress, airway hyperresponsiveness and tissue remodeling.	Malaviya, R., et al., Anti-TNFalpha therapy in inflammatory lung diseases. Pharmacol Ther, 2017. 180: p. 90-98.
**IL6**	Increased activation of T cell in BALF play an important role in the inflammatory response of acute and severe MPP. Il-6 can be used as an important predictor of the severity and prognosis of the disease.	Guo, L., et al., Increased T cell activation in BALF from children with Mycoplasma pneumoniae pneumonia. Pediatr Pulmonol, 2015. 50(8): p. 814-9.
**IL1β**	Activates T-cells and macrophages, a major pro-inflammatory cytokine that is involved in many important cellular functions such as proliferation, differentiation, and activation of different cell types.	Yang, J., et al., Interleukin-1beta responses to Mycoplasma pneumoniae infection are cell-type specific. Microb Pathog. 2003 Jan;34(1):17-25.
**MCP1**	MCP1 possesses proinflammatory properties and their biological effect can in part account for the persistance of inflammation in the bronchial mucosa of chronic bronchitis.	Rózyk, KJ., et al., Monocyte chemotactic and activating factor/monocyte chemoattractant protein in bronchoalveolar lavage fluid from patients with atopic asthma and chronic bronchitis. Relationship to lung function tests, bronchial hyper-responsiveness and cytology of bronchoalveolar lavage fluid. Immunol Lett. 1997 Jun;58(1):47-52.
**IL4**	A predominant TH2-like cytokine response in Mycoplasma pneumonia, thus representing a favorable condition for IgE production.	Koh, Y.Y., et al., Levels of interleukin-2, interferon-gamma, and interleukin-4 in bronchoalveolar lavage fluid from patients with Mycoplasma pneumonia: implication of tendency toward increased immunoglobulin E production. Pediatrics, 2001. 107(3): p. E39.
**IL10**	Potent suppressor of macrophage functions, down-regulates Th1 cytokines, enhances antibody production.	Commins S, et al., The extended IL-10 superfamily: IL-10, IL-19, IL-20, IL-22, IL-24, IL-26, IL-28, and IL-29. The Journal of allergy and clinical immunology (2008) 121(5):1108-11.
**IFNγ**	In most of the patients, lymphocytes produced a larger amount of IFN-gamma in the convalescent stage than in the acute stage, when lymphocytes were stimulated with M. pneumoniae antigen. In some patients, however, lymphocytes did not produce IFN-gamma during the course of illness. Such lymphocytes, negative for	Nakayama T, et al., Interferon production during the course of Mycoplasma pneumoniae infection. The Pediatric infectious disease journal (1992) 11(2):72-7.

	IFN-gamma production in response to M. pneumoniae, produced IFN-gamma after the depletion of macrophages, and readdition of macrophages suppressed the production of IFN-gamma by lymphocytes.	
**IL13**	IL13 is found to cause mucin overproduction through STAT6/EGFR-FOXA2 signaling and mucus plugging formation in MP infection, which results in pulmonary atelectasis or consolidation.	Hao Y, et al. Mycoplasma pneumoniae modulates STAT3-STAT6/EGFR-FOXA2 signaling to induce overexpression of airway mucins. Infection and immunity (2014) 82(12):5246-55.
**IL5**	Involved in the production, differentiation, maturation and activation of the eosinophils, stimulate the release of inflammatory mediators from epithelial cells, development of allergic airway inflammation.	Choi, I.S., et al., Increased serum interleukin-5 and vascular endothelial growth factor in children with acute mycoplasma pneumonia and wheeze. Pediatr Pulmonol, 2009. 44(5): p. 423-8.
**sCD40L**	Blocking CD40/CD40L interactions with blocking Ab decreased BAL production of Th1-mediators.	Zhang-Hoover J, et al., CD40/CD40 ligand interactions are critical for elicitation of autoimmune-mediated fibrosis in the lung. Journal of immunology (2001) 166(5):3556-63.
**Flt3L**	Provide the key link between innate and adaptive immunity by recognizing pathogens and priming pathogen-specific immune responses.	Sathaliyawala T, et al. Mammalian target of rapamycin controls dendritic cell development downstream of Flt3 ligand signaling. Immunity (2010) 33(4):597-606.
**IL2**	Promotes the maturation of primitive T cells, regulates endogenous T regulator cells, which suppress airway inflammation.	Koh, Y.Y., et al., Levels of interleukin-2, interferon-gamma, and interleukin-4 in bronchoalveolar lavage fluid from patients with Mycoplasma pneumonia: implication of tendency toward increased immunoglobulin E production. Pediatrics, 2001. 107(3): p. E39.
**IFNα2**	Mycoplasma pneumoniae induces interferon-alpha to participate in membrane interaction	Capobianchi MR, Lorino G, et al. Membrane interactions involved in the induction of interferon-alpha by Mycoplasma pneumoniae. Antiviral research (1987) 8(3):115-24.
