# Supplementary material for: Cytokine signatures associate with disease severity in children with Mycoplasma pneumoniae pneumonia
Source: Sci Rep. 2019 Nov 28;9:17853. doi: 10.1038/s41598-019-54313-9 (PMC6882793; doi:10.1038/s41598-019-54313-9)
Supplement: Supplementary file 3 — Dataset 3 [file 41598_2019_54313_MOESM3_ESM.pdf]

Title: Cytokine signatures associate with disease severity in children with *Mycoplasma pneumoniae* pneumonia

Mingyue Yang[1], Fanzheng Meng[2], Man Gao[2], Genhong Cheng[3], Xiaosong Wang[1*]

* Correspondence: Xiaosong Wang

Postal address: No. 519 Dongminzhu Street, Chaoyang District, Changchun 130021, China

Tel: +86 138 4498 9650

Fax: +86 431 8565 4528

E-Mail: xiaosongwang@jlu.edu.cn

1. Department of Translational Medicine, the First Hospital of Jilin University, Changchun, China
2. Department of Pediatrics, the First Hospital of Jilin University, Changchun, China.
3. Department of Microbiology Immunology and Molecular Genetics, University of California Los Angeles, Los Angeles, USA

Table S3. The accuracy and precision of the kit for Luminex assays

	% Recovery in matrix	Intra-assay precision % CV	Inter-assay precision % CV
**TNFα**	97.8	2.6	13.0
**IL6**	96.1	2.0	18.3
**IL1β**	94.9	2.3	6.7
**MCP1**	98.3	1.5	7.9
**IL4**	94.5	2.9	14.2
**IL10**	97.7	1.6	16.8
**IFNγ**	98.1	1.6	12.0
**IL13**	95.0	2.2	9.2
**IL5**	99.9	2.6	10.8
**sCD40L**	95.2	3.7	18.9
**Flt3L**	98.2	2.4	6.6
**IL2**	95.4	2.1	6.3
**IFNα2**	93.9	2.4	13.3
